# Association of Candidate Genes With Submergence Response in Perennial Ryegrass

**DOI:** 10.3389/fpls.2017.00791

**Published:** 2017-05-16

**Authors:** Xicheng Wang, Yiwei Jiang, Xiongwei Zhao, Xin Song, Xiangye Xiao, Zhongyou Pei, Huifen Liu

**Affiliations:** ^1^Institute of Pomology, Jiangsu Academy of Agricultural Sciences and Jiangsu Key Laboratory for Horticultural Crop Genetic ImprovementNanjing, China; ^2^College of Agronomy and Resources and Environment, Tianjin Agricultural UniversityTianjin, China; ^3^Department of Agronomy, Purdue University, West LafayetteIN, USA; ^4^Department of Crop Genetics and Breeding, Sichuan Agricultural UniversityChengdu, China; ^5^College of Pastoral Agriculture Science and Technology, Lanzhou UniversityLanzhou, China

**Keywords:** association mapping, candidate gene, *Lolium perenne*, recovery, submergence

## Abstract

Perennial ryegrass is a popular cool-season grass species due to its high quality for forage and turf. The objective of this study was to identify associations of candidate genes with growth and physiological traits to submergence stress and recovery after de-submergence in a global collection of 94 perennial ryegrass accessions. Accessions varied largely in leaf color, plant height (HT), leaf fresh weight (LFW), leaf dry weight (LDW), and chlorophyll fluorescence (Fv/Fm) at 7 days of submergence and in HT, LFW and LDW at 7 days of recovery in two experiments. Among 26 candidate genes tested by various models, single nucleotide polymorphisms (SNPs) in 10 genes showed significant associations with traits including 16 associations for control, 10 for submergence, and 8 for recovery. Under submergence, *Lp1-SST* encoding sucrose:sucrose 1-fructosyltransferase and *LpGA20ox* encoding gibberellin 20-oxidase were associated with LFW and LDW, and *LpACO1* encoding 1-aminocyclopropane-1-carboxylic acid oxidase was associated with LFW. Associations between *Lp1-SST* and HT, *Lp6G-FFT* encoding fructan:fructan 6G-fructosyltransferase and Fv/Fm, *LpCAT* encoding catalase and HT were also detected under submergence stress. Upon de-submergence, *Lp1-SST, Lp6G-FFT*, and *LpPIP1* encoding plasma membrane intrinsic protein type 1 were associated with LFW or LDW, while *LpCBF1b* encoding C-repeat binding factor were associated with HT. Nine significant SNPs in *Lp1-SST, Lp6G-FFT, LpCAT*, and *LpACO1* resulted in amino acid changes with five substitutions found in *Lp1-SST* under submergence or recovery. The results indicated that allelic diversity in genes involved in carbohydrate and antioxidant metabolism, ethylene and gibberellin biosynthesis, and transcript factor could contribute to growth variations in perennial ryegrass under submergence stress and recovery after de-submergence.

## Introduction

Plant species or cultivars differ in growth responses to submergence stress. Escape or quiescence can be a strategy for plant survival from submergence stress (Bailey-Serres and Voesenek, 2008). Escape type plants show rapid shoot elongation to potentially grow above the water and re-stablish air contact. In contrast, quiescence type plants conserve energy by minimizing shoot elongation under the water, allowing plants to generate new tissues after de-submergence (Perata and Voesenek, 2007; Colmer and Voesenek, 2009). The severity and duration of stress, plant species and cultivars, and variation of environmental conditions all influence submergence tolerance, as well as the capability of plant regrowth following de-submergence.

Numerous physiological and molecular alterations are involved in genotypic variations in response to submergence stress and recovery (Vashisht et al., 2011; Yu et al., 2012; Van Veen et al., 2013; Campbell et al., 2015). Carbohydrate consumption and conservation play an important role in stress tolerance, and depletion of carbohydrates under submergence stress largely influenced plant growth and survival (Setter and Laureles, 1996; Sasidharan et al., 2013; Liu and Jiang, 2016). In C3 cool-season temperate grasses, fructan is a major component of non-structural carbohydrate reserve (Chatterton et al., 1989). Decreases in fructan and total water soluble carbohydrate (WSC) content were found in perennial ryegrass exposed to submergence stress, but the tolerant accession had relatively higher levels of fructan and WSC than the intolerant one (Yu et al., 2012). WSC and fructan contents decreased to a similar level in tolerant species of alligatorweed under submergence, but de-submerged plants showed rapid recovery of carbohydrate, which was independent from stored carbohydrate reserves at the starting point of recovery (Ye et al., 2016). It seems that both carbohydrate utilization at the end of submergence and recovery of photosynthesis after de-submergence could be associated with the rate of regrowth (Luo et al., 2011; Yu et al., 2012).

Submergence-induced plant growth alterations are also mediated by phytohormone interactions (Voesenek et al., 2003; Tamang and Fukao, 2015). Under hypoxic conditions, the activities of ethylene biosynthetic enzymes were stimulated, causing increased ethylene levels (Sasidharan and Voesenek, 2015). In rice, an increase in the level of endogenous ethylene and ethylene-mediated gibberellin biosynthesis were observed in both escape and quiescence types in response to submergence stress (Tamang and Fukao, 2015). Through interactions between ethylene, brassinosteroids (BR), *SUB1A* and *SKs*, gibberellin-mediated shoot elongation either increased to grow out of the water (escape) or was suppressed for carbohydrate conservation during submergence (quiescence). The stimulated elongation of petioles under submergence stress was associated with accumulated ethylene in submerged petioles, a fast and substantial decrease of the endogenous abscisic acid (ABA) concentration, and a certain level of endogenous auxin and gibberellin in marsh dock (Voesenek et al., 2003). Similar results in lotus demonstrated that the increased ethylene altered the balance between ABA and GA, which contributed to the submergence-induced petiole elongation (Jin et al., 2017).

Antioxidant metabolisms may promote submergence tolerance and recovery upon reoxygenation in plants (Tan et al., 2010; Liu and Jiang, 2015, 2016). The increased shoot activities of catalase (CAT) and peroxidase (POD) were more pronounced in relatively slow-growing genotypes, while greater reductions in root activities of superoxide dismutase (SOD), CAT, POD and ascorbate peroxidase (APX) treatments and increased malondialdehyde concentrations were found in the fast growing genotypes of perennial ryegrass (Liu and Jiang, 2015). Submergence also decreased activities of SOD and APX but increased CAT and POD activities in two creeping bentgrass cultivars (Liu and Jiang, 2016). Moreover, reoxygenation after de-submergence can cause oxidative injury due to increased oxygen uptake and accelerated mitochondrial activities, potentially leading to lipid peroxidation and membrane leakage (Blokhina et al., 2003). A study by Yuan et al. (2017) found that the Jasmonate-inducible accumulation of antioxidants may alleviate oxidative damage caused by reoxygenation in *Arabidopsis thaliana*, improving plant survival after submergence. The results suggest activation of the antioxidant defense system by the reoxygenation process could reduce oxidative damage and maintain cellular redox homeostasis (Biemelt et al., 1998; Skutnik and Rychter, 2009).

Transcriptional profiling of genotypes contrasting submergence tolerance revealed numerous genes in response to submergence stress (Tamang et al., 2014; Campbell et al., 2015; Chen et al., 2016; Rivera-Contreras et al., 2016). A comparison of a fast-growing escape type of marsh dock with a slow-growing quiescence type of common sorrel identified molecular processes related to carbon starvation, toxins, and ion homeostasis that explained the adaptive growth differences in these two species (Van Veen et al., 2013). Root transcript profiling showed that glycolysis and fermentation genes and a gene encoding sucrose synthase were more strongly induced in less submergence tolerant great yellowcress than in tolerant creeping yellowcress (Sasidharan et al., 2013). Upon de-submergence, a large portion of reoxygenation-responsive genes were identified but also significantly overlapped with submergence-responsive genes in soybean plants (Tamang et al., 2014). The results indicate the induction or down-regulation of certain genes is associated with plant responses during and after submergence stress. These genes could be core conserved, genotype- and organ-specific in fulfilling their role in mediating submergence responses of plants (Tamang et al., 2014; Van Veen et al., 2016). Through genome-wide association analysis combined with biparental QTL mapping approaches, candidate gene *HXK6* encoding a hexokinase was identified, with its role in mediating seed germination and contributing to the differences in coleoptile growth between Japonica and Indica varieties under submerged condition (Hsu and Tung, 2015). In *Arabidopsis thaliana*, genome wide association analysis on 81 natural accessions detected 77 genes (within 10 kb of the associated SNP markers) significantly associated with submergence response and involved in many physiological processes including carbon starvation and fermentation (Vashisht et al., 2016). These genes are important regulators for controlling diverse plant responses to submerged conditions.

Although a large number of genes have been identified in plants under submergence stress, little is known about whether allelic diversities of candidate genes involved in carbohydrate, hormone regulation and antioxidant metabolism cause variations in plant growth under submergence stress and recovery after de-submergence, especially in perennial grass species. Perennial ryegrass is one of the most important cool-season turf and forage grasses. This species has a diverse germplasm, diploid genetics, and more available genomic resources (Studer et al., 2012; Byrne et al., 2015) than other major economically important perennial forage and turf grass species, thus providing a good model for studying the genetic basis of submergence tolerance. We designed the experiment to identify associations of candidate genes with growth and physiological traits to submergence stress and recovery after de-submergence in perennial ryegrass. The knowledge gained from this study will reveal genetic mechanisms of submergence tolerance. The results would benefit germplasm enhancement in perennial ryegrass as well as in other major cool-season perennial grass species with more complex genomes.

## Materials and Methods

### Plant Materials and Growing Conditions

**FIGURE 1:**
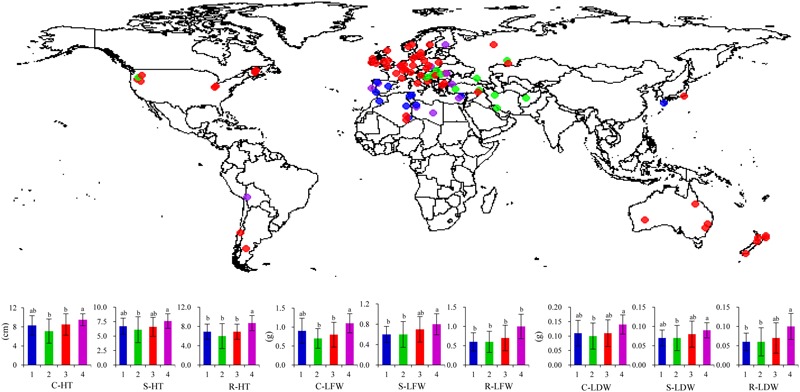
**Germplasm origin and population structure-affected traits among worldwide perennial ryegrass accessions.** Accessions are color-coded by subgroup type identified previously based on population structure (Yu et al., 2011). Accessions with the same color belong to the same subgroup. HT, plant height; LFW, leaf fresh weight; LDW, leaf dry weight; C, S, and R represent the non-stress control, submergence, and recovery after de-submergence, respectively. Means followed by same letter are not significantly different at *P* < 0.05 within a trait for a given subgroup (column). Bars indicate standard deviation.

A global collection of 94 perennial ryegrass accessions was used in this study including 30 wild, 33 cultivars or cultivated, and 31 uncertain materials according to the germplasm bank classification (Yu et al., 2011). The selection of plant materials was based on geographical locations of accessions to maximize ecotype diversity (**Figure 1** and Supplemental Table S1). A single seed from each accession was sown in a greenhouse into a plastic pot (4-cm diameter, 9-cm deep) containing a sandy-loam soil with a pH of 6.9. Each accession was propagated through tillers multiple times to ensure genetic uniformity. Two experiments (Experiments 1 and 2) were conducted in a greenhouse in 2009 and 2010 using newly propagated plants (Yu et al., 2012). The duration of Experiment 1 was from October 6 to November 21, 2009, and from December 23, 2009 to February 5, 2010 for Experiment 2. Detailed information of environmental conditions during plant growth was described previously (Yu et al., 2012). Prior to submergence treatment, all the plants were cut to about 5–6 cm above the soil surface to obtain a uniform height.

### Submergence Treatment and Recovery

Submergence stress was imposed by submerging the grass pots in (86 cm length × 38 cm width × 30 cm height) plastic containers with tap water kept at 5 cm above the top of the grasses. The stress treatments began on November 14 of 2009 for Experiment 1 and were repeated on January 29 of 2010 for Experiment 2, lasting 7 days for both. After 7 days of stress, the submerged plants were taken out of the water to allow recovery for 7 days. The environmental conditions during the treatment period were described previously (Yu et al., 2012).

### Phenotypic Traits

Leaf color, plant height (HT), leaf fresh weight (LFW), leaf dry weight (LDW), and chlorophyll fluorescence (Fv/Fm) were assessed for indications of plant growth and physiological responses to submergence stress. Leaf color was visually rated on a scale of 1 (yellow) to 9 (dark green). Grass were cut to a similar height prior to stress, the measured HT referred to plant growth occurring only during the 7-day treatments. Leaves corresponding to this HT were cut as measurement of LFW, and LDW was determined after the tissues were dried in an oven at 80°C for 3 days. Fv/Fm was determined in the dark on randomly selected leaves using a fluorescent meter (OS-30P, OPTI-Sciences, Hudson, NH, USA). Only HT, LFW and LDW were evaluated after de-submergence, but not leaf color and Fv/Fm due to minimum differences in these two measurements for both experiments.

### Experimental Design and Repeatability of Traits

The experiment was a split plot design with three replications for both experiments (Yu et al., 2012). The main plot was the submergence treatment and the subplot was the set of accessions. The grass pots were completely randomly assigned into containers within treatment regimes, respectively. The repeatability of phenotypic traits across two experiments as reflected by heritability (*h*^2^) was calculated using PROC MIXED (SAS Institute, Version 9.1, Cary, NC, USA). The *h*^2^ was calculated as follows:

$$h^{2}{}^{\;}=\;\sigma_{\text{g}}^{\text{2}}/(\sigma_{\text{g}}^{\text{2}}+\sigma_{\text{ge}}^{\text{2}}/l\;{+}^{\;}\sigma_{\text{e}}^{\text{2}}/rl),$$

where $\sigma_{e}^{2}$ is the variance component for genotypes, σ^2^_ge_ for genotype-by-environment, $\sigma_{e}^{2}$ for error; *r* is the number of replications, and *l* is number of environment. Based on the outcome of *h*^2^, least square means were estimated for each accession across two experiments.

### Genotyping

The population was genotyped by using 109 published genome-wide simple sequence repeat markers in perennial ryegrass (Kubik et al., 2001; Jensen et al., 2005; Gill et al., 2006; King et al., 2008). The detailed procedures of DNA extraction, PCR amplification, and allele identification for this population were described previously (Yu et al., 2011). Population structure (Q) was determined by using STRUCTURE 2.3.2 software (Pritchard et al., 2000) and pairwise relative kinship (K) was determined using SPAGeDi (Hardy and Vekemans, 2002) and both were assessed previously by Yu et al. (2011).

### Gene Sequencing and SNP Identification

Twenty-six candidate genes in the functions of gibberellic acid biosynthesis, ethylene biosynthesis, kinase, dehydration protection, aquaporin, transcription factor, and anaerobic, carbohydrate and antioxidant metabolisms related to growth and submergence tolerance were selected for sequencing. Of them, sequences of genes involved in antioxidant metabolism (*LpCAT, LpChl Cu-ZnSOD, LpCyt Cu-ZnSOD, LpGPX, LpMnSOD, LpFeSOD, LpAPX, LpMDAR, LpDHAR, LpGR*), kinase (*LpMAPK*), dehydration (*LpLEA3*), aquaporin (*LpPIP1* and *LpTIP1*), and transcription factor (*LpCBF1b, LpCBF3b, LpCBF3c*, and *LpCB4b*) for this perennial ryegrass population were obtained previously (Yu et al., 2013, 2015). Additional candidate genes including gene bank accession AY014277.1 for *GA20ox*, AY551432.1 for *GA2ox4*, AM407402.1 for *1-SST*, AM407401 for *6G-FFT*, AM407403.1 for *6-SFT*, XM_003573365.3 for *LDH*, XM_003580260.1 for *PDC*, Bradi4g31820 for *ACO1*, and Bradi1g10030.1 for *ACS* were sequenced in the population (**Table 2**).

Direct genomic DNA sequencing often results in unclean sequence readings due to the high outcrossing and heterozygous nature of perennial ryegrass, thus, the more problematic introns were excluded for sequencing. We designed primers based on a single long exon for these genes (Supplementary Table S2) and genome DNA was used as the PCR template. Sequencing reactions were performed using Big Dye Terminator kit version 3.1 (Applied Biosystems, Carlsbad, CA, USA) and sequencing was conducted using an ABI 3730 genetic analyzer according to the manufacturer’s instructions (Applied Biosystems, Carlsbad, CA, USA) in the Genomic Center at Purdue University. SNPs were identified using the NovoSNP program 3.0.1 Microsoft Windows Platform version (Weckx et al., 2005).

### Nucleotide Diversity and Linkage Disequilibrium (LD)

The nucleotide diversity (π), nucleotide polymorphism (𝜃), and Tajima’s *D* were analyzed with SNPs for each gene using TASSEL (Bradbury et al., 2007). The LD was also calculated for candidate genes using TASSEL. The generated *r*^2^ value was plotted against the physical distance among each pair of SNPs. Overall LD was generated by pooling data from all candidate genes.

### Association Analysis

Quantile-quantile (Q-Q) plots for model comparisons of simple linear (S), Q, K and Q + K across all traits were analyzed using R, and the best fit model was selected for association analysis of each trait. Associations between candidate genes and traits of leaf color, HT, LFW, LDW, and Fv/Fm were analyzed using TASSEL 2.1 software with the following three data set: (1) well-drained control (C); (2) submergence stress (S); (3) Recovery after de-submergence (R). Minor alleles with frequency < 5% were removed prior to association analysis. Associations were considered to be significant only at a *P*-value lower than the *P*_threshold_-value, calculated using *P*_threshold_ = 0.05/N, where N was the number of SNPs in a candidate gene.

### Functional Substitution and Phylogenetic Tree

The putative functional amino acid substitutions were analyzed only for genes significantly associated with traits. A full length gene sequence was obtained from NCBI or from perennial ryegrass genome assembly (Byrne et al., 2015) for anchoring SNP positions in each gene. Amino acid sequences of each peptide were then inferred and compared using NCBI to identify amino acid substitutions. A phylogenetic tree was constructed in MEGA 6 (Tamura et al., 2013), incorporating the Neighbor-Joining method (Saitou and Nei, 1987) with bootstrap analysis of 1000 replicates. The species used for constructing the Neighbor-Joining Tree included perennial ryegrass, tall fescue, *Brachypodium distachyon*, wheat, barley, rye, maize, rice, oats, and foxtail millet.

## Results

### Trait Variations and Repeatability

For all accessions across two experiments, large variations in traits were observed under the control, submergence stress, and recovery periods (**Table 1**). Specifically, C-color ranged from 3.7 to 9.0 with a mean of 6.0 and S-Color ranged from 1.7 to 6.8 with a mean of 3.5. Fv/Fm varied from 0.71 to 0.82 for the control plants and from 0.66 to 0.81 for the submerged plants. The minimum and maximum HT were 2.0- and 16.3-cm for control, 1.44-and 11.7-cm for submergence, and 0.77- and 11.6-cm for recovery, respectively. LFW and LDW spanned from 0.34- to 1.7-g and 0.04- to 0.23-g for the control, 0.19- to 1.57-g and 0.01- to 0.19-g for submergence, and 0.17- to 1.62-g and 0.02- to 0.17-g for recovery, respectively.

**Table 1:** Range and mean values, percentage of variation, and repeatability of leaf color (color), plant height (HT), chlorophyll fluorescence (Fv/Fm), leaf fresh weight (LFW), leaf dry weight (LDW) under non-stress control (C), submergence stress (S), and recovery after de-submergence (R) in 94 perennial ryegrass accessions across two experiments.

Traits	Minimum	Maximum	Mean	Variation (%)	Repeatability
C-Color	3.73	9.00	5.98	58.6	0.85
C-HT (cm)	2.01	16.3	8.42	87.6	0.76
C-Fv/Fm	0.78	0.82	0.81	4.88	0.67
C-LFW (g)	0.34	1.70	0.86	79.9	0.61
C-LDW (g)	0.04	0.23	0.11	84.6	0.55
S-Color	1.67	6.83	3.51	75.6	0.60
S-HT (cm)	1.45	11.7	6.69	87.7	0.85
S-Fv/Fm	0.66	0.81	0.76	18.4	0.46
S-LFW (g)	0.19	1.57	0.66	87.9	0.64
S-LDW (g)	0.01	0.19	0.08	93.5	0.58
R-HT (cm)	0.77	11.6	7.00	93.4	0.78
R-LFW (g)	0.17	1.62	0.68	89.5	0.58
R-LDW (g)	0.02	0.17	0.07	89.7	0.57

Repeatability (*h*^2^) was calculated across two experiments. The *h*^2^ values of all traits were higher than 0.5 except for S-Fv/Fm (**Table 1**). The average *h*^2^ for traits under control, submergence, and recovery were 0.69, 0.62, and 0.65, respectively. The high repeatability over testing two environments allowed least square means (LSmeans) of individual traits to be calculated and used for association analyses of genes and traits.

### Traits Within Population Structure

Four population structures (subgroups) were identified previously in this population with no obvious kinship (Yu et al., 2011). There were 11, 12, 60, and 11 accessions from subgroups 1–4, respectively (**Figure 1**). Subgroup 1 contained accessions mainly from southern Europe and northern Africa, and subgroup 2 consisted of accessions mainly from eastern Europe and western Asia. Subgroup 3 was the largest one with more diverse geographic locations mainly including accessions from northern Europe, western Europe, USA, Canada, Australia, and New Zealand. Accessions from subgroup 4 showed mixed geographic locations from northern Africa, eastern and southern Europe. The average values of all traits differed in four subgroups except for C-Fv/Fm, S-Fv/Fm, and S-color (**Figure 1**). On average, C-color was 5.3, 6.6, 6.2, and 5.0 in subgroup 1, 2, 3, and 4, respectively. Generally, the highest values of HT, LFW, and LDW were found in subgroup 4 and the lowest in subgroup 2 (**Figure 1**). No differences in traits were noted between subgroup 1 and 3. The separation in trait values of subgroups could be related to geographical locations of accessions.

### Nucleotide Diversity, Linkage Disequilibrium and SNP Number

**FIGURE 2:**
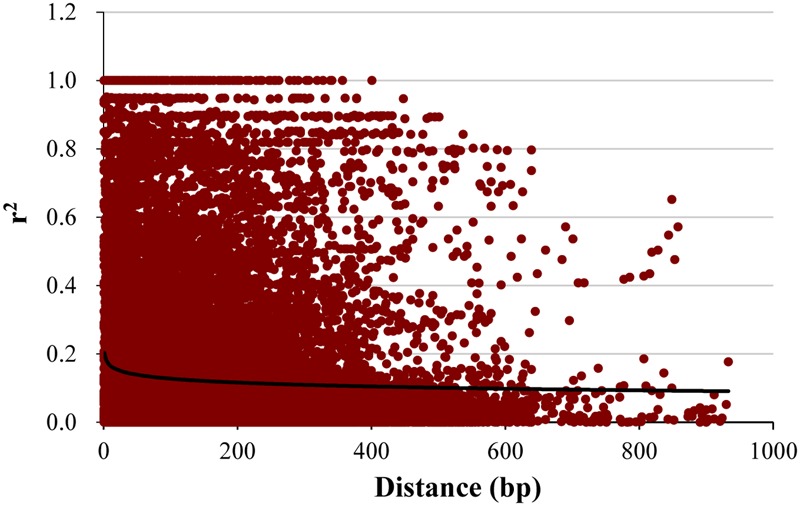
**Linkage disequilibrium (LD) decay in perennial ryegrass.** Plots of squared correlations of allele frequencies (*r*^2^) against physical distance between pairs of SNPs in the pooled 26 genes.

Across 26 candidate genes, the highest value of nucleotide diversity (π) was found in *LpPIP1* (0.34) and the lowest in *LpCBF1b* (0.004) with a mean value of 0.091 (**Table 2**). Watterson’s 𝜃_w_ ranged from 0.0086 for *LpCBF1b* to 0.15 for *LpCBF3c* with a mean value of 0.042 (**Table 2**). Positive Tajima’s *D* values were seen in all genes except for *LpCBF1b, LpCBF3c*, and *LpGPX*. No Tajima’s *D* was detected in *LpCBF3b, LpMAPK*, and *LpMnSOD*. For the LD pattern, the mean r^2^ for the pair of SNPs with all candidate genes was 0.16, ranging from 0.034 (*LpCBF3c*) to 0.48 (*LpCBF1b*) (**Table 2**). Overall, a rapid LD decay was shown in perennial ryegrass across all genes (**Figure 2**). LD decay decreasingly extended to more than 900 kb within *r*^2^ = 0.1. Within the sequencing length, a total of 863 SNPs were detected in all genes, ranging from 2 (*LpCBF3b*) to 121 (*LpACO1*) after minor SNPs (<5%) were removed (**Table 2**). The average SNP frequency was 1/48.3 bp, ranging from 1/5 bp in *Lp1-SST* to 1/357 bp in *LpCBF3b*.

**Table 2:** Summary of genes used in this study, the number of single nucleotide polymorphism (SNP) sites, nucleotide diversity (*π*), nucleotide polymorphism (*𝜃*), linkage disequilibrium (LD), and Tajima’s *D* in each gene across a diverse perennial ryegrass population.

Gene	Full name	*π*	*𝜃*	Tajima’s *D*	LD	L_1_/bp	L_2_/bp	SNP	Freq
*LpACO1*	1-aminocyclopropane-1-carboxylicacid oxidase	0.087	0.035	4.90	0.050	768	736	121	6
*LpACS*	1-aminocyclopropane-1-carboxylic acid synthase	0.013	0.005	1.75	0.098	572	555	41	14
*Lp6G-FFT*	Fructan: fructan 6G-fructosyltransferase	0.032	0.021	1.44	0.062	793	757	51	15
*LpGA2ox4*	Gibberellin 2-oxidase 4	0.049	0.022	2.53	0.101	514	496	31	16
*LpGA20ox*	Gibberellin 20-oxidase	0.032	0.015	2.00	0.058	564	530	30	18
*LpLDH*	L-lactate dehydrogenase	0.029	0.017	1.84	0.207	613	586	70	8
*LpPDC*	Pyruvate dehydrogenase E1 component subunit alpha-3	0.028	0.013	2.74	0.065	587	545	57	10
*Lp6-SFT*	Sucrose: fructan 6-fructosyltransferase	0.107	0.055	2.27	0.272	500	472	20	24
*Lp1-SST*	Sucrose: sucrose 1-fructosyltransferase	0.151	0.091	2.18	0.114	527	498	109	5
*LpCAT*	Catalase	0.051	0.022	3.03	0.261	577	505	40	13
*LpCBF1b*	C-repeat binding factor 1b	0.004	0.009	-0.76	0.478	584	532	28	19
*LpCBF3b*	C-repeat binding factor 3b	0	0	?	0.001	808	714	2	357
*LpCBF3c*	C-repeat binding factor 3c	0.115	0.154	-0.47	0.034	943	720	3	240
*LpCBF4b*	C-repeat binding factor 4b	0.117	0.072	1.37	0.424	895	782	12	65
*LpChl Cu-Zn SOD*	Chloroplastic copper-zinc superoxide dismutase	0.175	0.064	4.41	0.095	428	400	21	19
*LpCyt Cu-Zn SOD*	Cytosolic copper–zinc superoxide dismutase	0.099	0.017	7.63	0.172	421	355	15	24
*LpDHAR*	Dehydroascorbate reductase	0.106	0.039	2.46	0.180	525	506	6	84
*LpFeSOD*	Iron superoxide dismutase	0.108	0.039	4.54	0.128	550	537	21	26
*LpGPX*	Glutathione peroxidase	0.074	0.104	-0.74	0.204	545	446	13	34
*LpGR*	Glutathione reductase	0.200	0.052	8.52	0.138	1130	1087	42	26
*LpLEA3*	Late embryogenesis abundant, group 3	0.159	0.069	3.55	0.111	446	350	26	13
*LpMAPK*	Mitogen-activated protein kinase	0	0	?	0.183	612	522	4	131
*LpMDHAR*	Monodehydroascorbate reductase	0.101	0.020	10.30	0.446	930	780	50	16
*LpMnSOD*	Manganese superoxide dismutase	0	0	?	0.043	289	245	13	19
*LpPIP1*	Plasma membrane intrinsic protein, type 1	0.340	0.087	8.48	0.138	544	495	20	25
*LpTIP1*	Tonoplast intrinsic protein, type 1	0.179	0.058	5.22	0.050	579	505	17	30

*L_*1*_, total length covered by the primer(s) designed in this study; L_*2*_, final readable sequenced length. Frequency of SNP, calculated by L_*2*_/SNP, stands for the average SNP per bp length.*

### Gene-Trait Association

**FIGURE 3:**
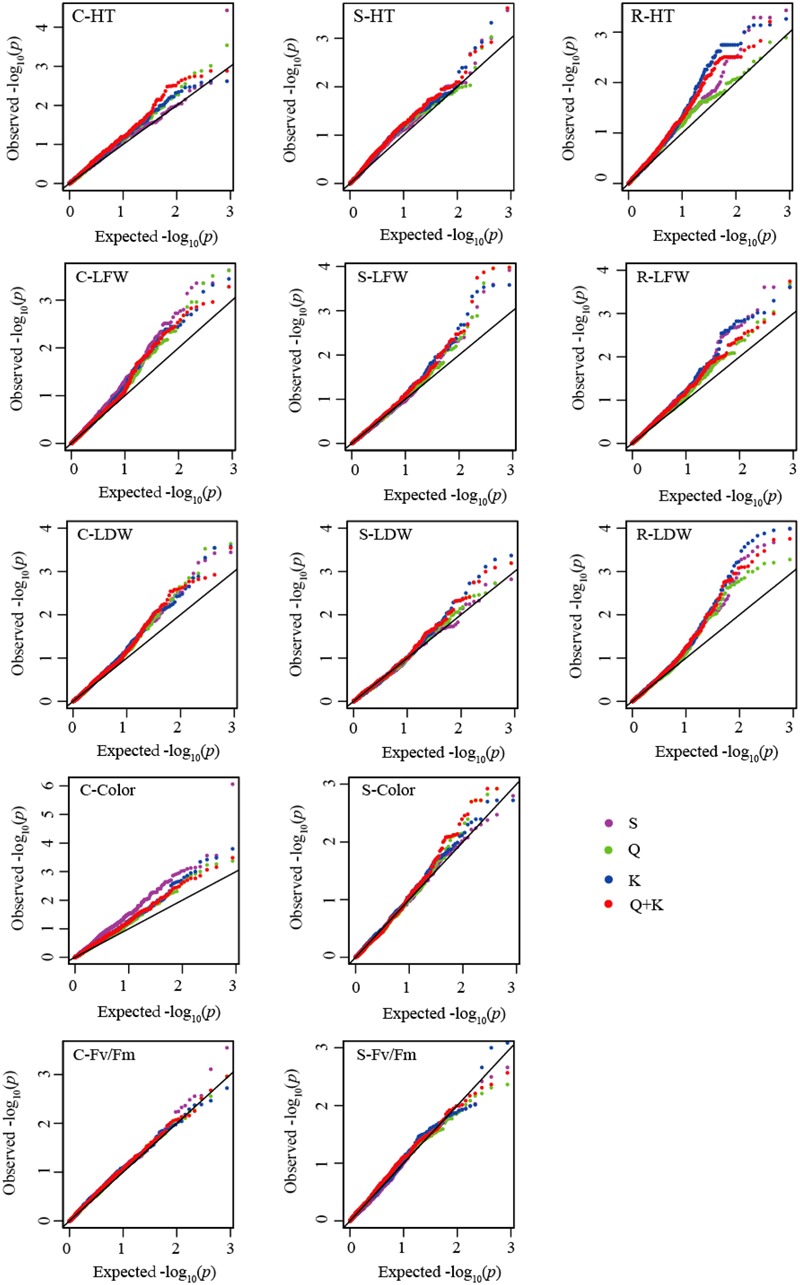
**Quantile-quantile (QQ) plots for model comparisons with plant height (HT), leaf fresh weight (LFW), leaf dry weight (LDW), leaf color, and chlorophyll fluorescence (Fv/Fm) under non-stress control (C), submergence stress (S), and recovery after de-submergence (R).** Leaf color and Fv/Fm were not collected after recovery, so that QQ plots were omitted for these two traits. The solid diagonal lines represent agreement between the observed and expected –log _10_ (*P*) for associations of 26 genes with traits. Color lines represent agreement between the observed and expected –log 10 (*P*) value for gene-trait associations analyzed with simple liner (S), population structure (Q), relative kinship (K), and Q + K implemented model, respectively.

Quantile-quantile plots verified the adequate model for controlling false positives for each gene-trait association for the control, submergence, and recovery periods (**Figure 3**). The results between the observed and expected -log _10_ (*P*) for associations of 26 genes with traits showed that either the S, Q, K or Q + K implemented model was suitable for analyzing gene-trait associations, depending on individual traits.

Through model testing mentioned above, 34 SNPs from 10 candidate genes exhibited significant associations with traits, including 16 SNPs for control, 10 for submergence, and 8 for recovery (**Table 3**, Supplementary Table S3). Multiple SNP sites in one particular gene were associated with different traits. For the control, SNPs from *Lp1-SST, Lp6G-FFT, LpCBF1b, LpACO1, LpFeSOD, LpACS*, and *Lpcyto Cu-Zn SOD* were associated with various traits. Under submergence, SNPs at position 1124 bp in *Lp1-SST* and at 1093 bp in *LpCAT* were associated with S-HT, and a SNP at 783 bp in *Lp6G-FFT* was associated with S-Fv/Fm. For S-LFW, significant associations were detected for SNPs at 909-, 1053-, 1087-, and 1124-bp in *Lp1-SST* and for a SNP at 469 bp in *LpACO1*. A SNP at 603 bp in *LpGA20ox* was associated with S-LFW and S-LDW under submergence. After de-submergence, a SNP at 1161 bp in *Lp1-SST* and a SNP at 267 bp in *LpCBF1b* were associated with R-HT. For *Lp1-SST*, SNPs at 871 bp was associated with R-LDW and SNP at 1091 bp associated with R-LFW and R-LDW. Significant associations were also found between SNPs at 835- and 938-bp in *Lp6G-FFT* and R-LFW and between a SNP at 1093 bp in *LpCAT* and R-LDW.

**Table 3:** Association of candidate genes with traits under submergence (S) and recovery (R) in 94 perennial ryegrass accessions.

Putative gene	Traits	SNP (bp)	Allele	*P*-value	Model
*Lp1-SST*	S-HT	1,124	C:CT:T	2.38E-04	Q + K
	S-LFW	1,053	A:AG:G	1.80E-04	Q + K
	S-LFW	909	C:CG:G	1.10E-04	Q + K
	S-LFW	1,087	G:GT	1.40E-04	Q + K
	S-LFW	1,124	C:CT:T	1.10E-04	Q + K
	R-HT	1,116	C:CG	9.00E-04	S
	R-LFW	1,091	C:CT:T	1.80E-04	Q + K
	R-LDW	1,091	C:CT:T	3.64E-05	Q
	R-LDW	871	T:GT	2.72E-05	Q
*Lp6G-FFT*	S-Fv/Fm	783	C:CT:T	8.16E-04	K
	R-LFW	835	G:GA:A	8.04E-04	Q + K
	R-LFW	938	A:AT:T	8.24E-04	Q + K
*LpACO1*	S-LFW	469	C:CT	4.13E-04	Q + K
*LpCBF1b*	R-HT	267	C:CT:T	3.70E-04	S
*LpGA20ox*	S-LFW	603	A:AG:G	6.20E-04	Q + K
	S-LDW	603	A:AG:G	1.00E-03	Q + K
*LpCAT*	S-HT	1,093	A:AG:G	3.67E-05	Q + K
*LpPIP*	R-LDW	273	C:CG:G	1.00E-03	Q

**FIGURE 4:**
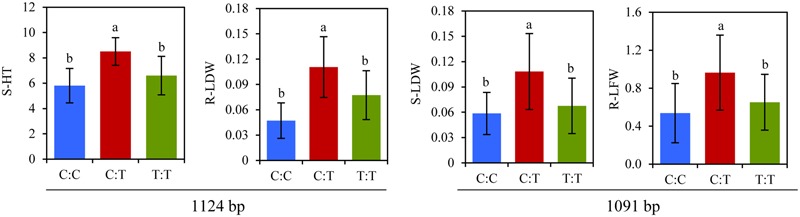
**Allelic variations of SNP at 1124 bp in *Lp1-SST* associated with plant height under submergence stress (S-HT) and leaf dry weight after recovery (R-LDW), and at 1091 bp associated with leaf fresh weight under submergence stress (S-LDW) and with leaf fresh weight after recovery (R-LFW) in perennial ryegrass accessions.** Columns with the same letter were not significantly different at *P* < 0.05. Bars indicate standard deviation.

Sequence variations and phenotypic differences of different alleles of *Lp1-SST* were compared under submergence or recovery after de-submergence (**Figure 4**). The mean values of S-HT and S-LDW under submergence at 1124 bp in *Lp1-SST* were significantly higher in the accessions carrying heterozygous C:T than the accessions carrying homozygous T:T and C:C. Similarly, at 1091 bp, accessions with heterozygous C:T had higher R-LFW and R-LDW after recovery than the accessions with homozygous T:T and C:C.

### Amino Acid Substitutions

The predicted amino acid substitutions were analyzed only for significant SNPs identified under submergence and recovery. Nine SNP positions in four genes resulted in amino acid changes (**Table 4**). Five SNPs at position 871-, 1087-, 1091-, 1116-, and 1124-bp in *Lp1-SST* had nucleotide substitutions from TCA to GCA, GCC to TCC, GTC to GCC, GAC to GAG, and CTG to CCG, causing amino acid substitutions from tyrosine (Y) to aspartic acid (D), alanine (A) to serine (S), valine (V) to A, D to Glutamic acid, (E) and leucine (L) to proline (P), respectively. A SNP at 835 bp in *Lp6G-FFT* showed one nucleotide substitution from GTC to ATC, resulting in an amino acid change from V to isoleucine (I), while a SNP at 938 bp had a substitution from TAC to TTC with the amino acid change from Y to phenylalanine (F). Nucleotide substitution also occurred at 1093 bp for *LpCAT* and 469 bp for *LpACO1*, leading to amino acid changes from V to methionine (M) and P to S, respectively.

**Table 4:** Amino acid substitution in the loci from candidate genes significantly associated with submergence and recovery traits.

Putative gene	SNP (bp)	Nucleotides	AA residual
*Lp1-SST*	871	TCA→GCA	Y/D
	1,087	GCC→TCC	A/S
	1,091	GTC→GCC	V/A
	1,116	GAC→GAG	D/E
	1,124	CTG→CCG	L/P
*Lp6G-FFT*	835	GTC→ATC	V/I
	938	TAC→TTC	Y/F
*LpCAT*	1,093	GTG→ATG	V/M
*LpACO1*	469	CCG→TCG	P/S

### Phylogenetic Analysis

**FIGURE 5:**
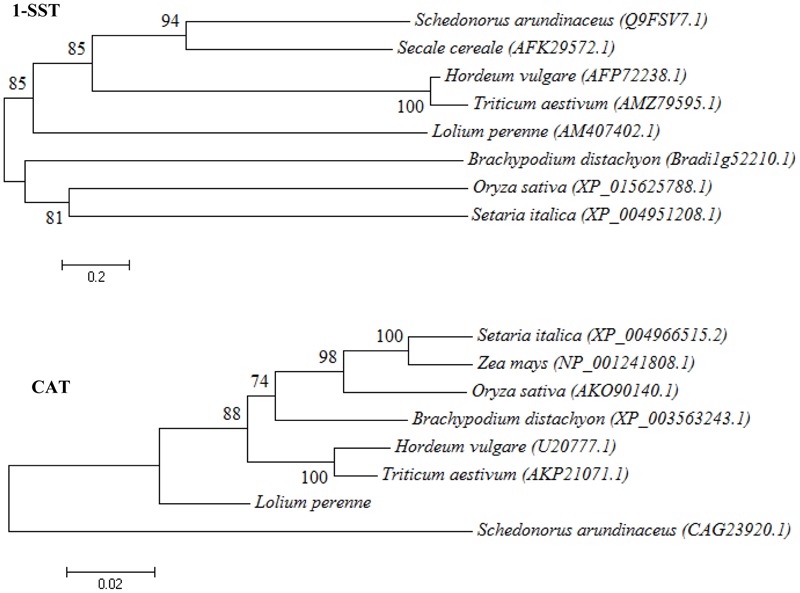
**Phylogenetic tree of sucrose: sucrose 1-fructosyltransferase (1-SST) and C-repeat-binding factor 1b (CBF1b) protein sequence from plant species.** NCBI protein accession numbers were used to indicate homologous protein.

Phylogenetic analysis of protein sequences in the selected species revealed that perennial ryegrass Lp1-SST was more closely related to that in wheat, barley, rye, and tall fescue than that in *Brachypodium distachyon*, rice and foxtail millet (**Figure 5**). Based on LpCAT sequences, perennial ryegrass was closer to wheat, barley, and *Brachypodium distachyon* than that in rice, maize, foxtail millet, and tall fescue (**Figure 5**). Lp6G-FFT in perennial ryegrass was more closely related to foxtail millet than that in Kentucky bluegrass, wheat, tall fescue, and barley (Supplementary Figure S1). LpACO1 in perennial ryegrass was more closely related to *Brachypodium distachyon* and foxtail millet and barley than maize, wheat, rice, and creeping bentgrass (Supplementary Figure S1).

## Discussion

Plant growth and physiological status are closely associated with plant survival from submergence stress. Perennial ryegrass accessions differed significantly in leaf color, Fv/Fm, HT, LFW, and LDW in response to submergence stress. Most of these traits were affected by population structure (**Figure 1**), indicating that structured traits may be related to a geographical origin of the accessions. Larger variations in these traits found in submerged plants more than in the control plants, except for HT, suggested a wide range of submergence response in the perennial ryegrass collection. The differences in HT among accessions may be due to their variation in natural growth habits, although submergence-induced elongation was observed in some perennial ryegrass accessions (Yu et al., 2012). In *Arabidopsis thaliana*, submergence tolerance was negatively correlated with underwater petiole elongation in 86 accessions (Vashisht et al., 2011). The results indicate complex responses of plants to submergence stress, influenced by many factors including the severity and duration of stress, plant growth status, as well as species and cultivars.

Natural variations in plant response to submergence stress provide an important basis for analyzing gene and trait associations. Carbohydrate metabolisms play a critical role in submergence tolerance. In temperate grasses, fructan biosynthesis is mainly controlled by fructosyltransferases (FTs) including sucrose:sucrose 1-fructosyltransferase (1-SST), fructan:fructan 1-fructosyltransferase (1-FFT), fructan:fructan 6G-fructosyltransferase (6G-FFT), and sucrose:fructan 6-fructosyltransferase (6-SFT) (Gallagher et al., 2015). 1-SST catalyzes the first reaction of the pathway. In this study, significant associations of SNPs at multiple sites in *Lp1-SST* with LFW, LDW, and HT under submergence as well as with LFW and RDW after recovery, demonstrated that allelic diversity of *Lp1-SST* contributed to diverse growth responses during or after stress (**Figure 4**). In particular, multiple amino acid substitutions caused by SNP changes indicated the importance of this gene in conferring submergence tolerance and recovery. Associations of *Lp6G-FFT* with S-Fv/Fm and with R-LFW after recovery were also identified, especially amino acid changes occurring at SNPs at 835 and 938 bp for R-LFW. The results indicated that *Lp1-SST* and *Lp6G-FFT* could influence fructan biosynthesis and assist in submergence tolerance and regrowth in perennial ryegrass after recovery from de-submergence.

Submergence enhances ethylene biosynthesis (Voesenek et al., 1993). 1-aminocyclopropane-1-carboxylic acid synthase (ACS) and 1-aminocyclopropane-1-carboxylic acid oxidase (ACO) are two key enzymes in ethylene biosynthesis (Yang and Hoffman, 1984). Previous studies showed that both ACS and ACO are involved in submergence-induced responses in shoot elongation (Vriezen et al., 1999; Zhou et al., 2002). When submerged, expression of *ACS1* and *ACO1* and proteins were all up-regulated in bog yellowcress (Vriezen et al., 1999), suggesting transcription and translation regulation of these two enzymes in the ethylene biosynthesis pathway. In rice, the transcript levels of most *OsACS* and *OsACO* family members were induced by submergence stress, but to a larger extent in the *OsACO* family than in the *OsACS* family, indicating function-related differences between these two gene families during submergence (Guo et al., 2015). Association of a SNP at 469 bp in *LpACO1* with R-LFW in this study supported the role of these two genes in mediating plant growth in perennial ryegrass. *ACO* is the last gene of the ethylene biosynthetic pathway. Particularly, the amino acid change from P to S caused by a SNP at 469 bp in *LpACO1* suggested its importance in regulating growth responses of perennial ryegrass to submergence stress.

Gibberellin (GA) regulates plant growth and development, including stem elongation and leaf expansion (Colebrook et al., 2014). GA20oxidase (GA20ox) catalyzes three steps from GA53 to GA20 and GA12 to GA9. Additionally, GA3oxidase (GA3ox) catalyzes the final step from GA20 to GA1 and GA9 to GA4 (Ayano et al., 2014). In *Arabidopsis thaliana, GA20ox1, 2*, and *3* have an important role in growth and fertility (Plackett et al., 2012). The mutation of *GA20ox1, 2*, and *3* causes severe dwarfism and sterility (Plackett et al., 2012), and overexpression of *GA20ox1* enhances plant growth (Nelissen et al., 2012). Deepwater rice cultivars differed in expression level of *OsGA20ox2* under submergence conditions, and internode elongation may be caused by GA1 and GA4 accumulation after induction of the *GA20ox* gene in plants under deepwater (Ayano et al., 2014). Our results in association of *GA20ox* with S-LFW and S-LDW in perennial ryegrass supported the observation of regulation of *GA20ox* on plant growth under submergence stress, although such association did not cause amino acid change.

The C-repeat-binding factor (CBF) genes encoding transcriptional activators control the expression of genes containing the C-repeat-dehydration responsive element DNA regulatory element in their promoters (Gilmour et al., 2000). There were 10 putative *CBF* genes in perennial ryegrass, similar to either the *HvCBF3* or *HvCBF4* subgroups in barley (Tamura and Yamada, 2007). Overexpression of *CBF* genes inhibited plant growth (Achard et al., 2008; Zhou et al., 2014), which may be due to inhibition of GA-dependent elongation growth. A co-expression network of *CBF* genes was induced by submergence stress but was more activated in the tolerant line of maize (Campbell et al., 2015). Four *LpCBF* genes were tested for their relationship with traits, but only *LpCBF1b* was significantly associated with C-LDW, C-HT, and R-HT. The results suggest a role of *LpCBF1b* on regrowth for perennial ryegrass plants following submergence stress.

Transcriptomic analysis of submergence-tolerant and sensitive *Brachypodium distachyon* ecotypes reveals oxidative stress as a major tolerance factor (Rivera-Contreras et al., 2016). Among antioxidant enzymes, SOD scavenges $O_{2}^{•–}$ to H_2_O_2_ (Bowler et al., 1992), and H_2_O_2_ can be decomposed by several pathways including the catalase (CAT) and ascorbate-glutathione cycles at different cellular locations (Mittler, 2002). The increased transcript levels of *SOD* or *CAT* were noted in maize (Zhang et al., 2009) and in creeping yellowcress under submergence stress (Sasidharan et al., 2013). The associations of *Lpcyto Cu-Zn SOD* and *LpCAT* with HT detected under non-stress or submergence indicated contributions of these genes to HT variations in perennial ryegrass accessions. In particular, a SNP at 1093 bp in *LpCAT* causing an amino acid change after recovery suggested a link between *LpCAT* and plant regrowth by potentially diminishing oxidative injury upon re-oxygenation.

## Conclusion

Significant associations were identified between candidate genes and growth and physiological traits in perennial ryegrass under the non-stress, submerged and recovery after de-submergence conditions. Allelic variations in *Lp1-SST, Lp6G-FFT, LpCAT*, and *LpACO1* caused amino acid substitutions, especially for *Lp1-SST* with multiple substitutions. The results suggest that allelic diversities of genes involved in carbohydrate and ethylene biosynthesis, antioxidant metabolism, and transcription factor may contribute to variable plant growth responses to submergence stress and recovery after stress in the perennial ryegrass population. This discovery illustrated an important genetic mechanism underlying submergence response, which will be valuable for further studies of functional and regulatory genes involved in submergence tolerance in perennial ryegrass or other perennial grass species with a more complex genome.

## Author Contributions

XW conducted gene sequence and wrote the manuscript; YJ designed the experiments and led analyzing data and writing of the manuscript; XW, XZ, XS, XX, ZP, and HL analyzed data. All authors approved the manuscript.

## Conflict of Interest Statement

The authors declare that the research was conducted in the absence of any commercial or financial relationships that could be construed as a potential conflict of interest.

## References

[B1] Achard P. Gong F. Cheminant S. Alioua M. Hedden P. Genschik P. (2008). The cold-inducible CBF1 factor–dependent signaling pathway modulates the accumulation of the growth-repressing DELLA proteins via its effect on gibberellin metabolism. *Plant Cell* 20 2117–2129. 10.1105/tpc.108.058941 18757556 PMC2553604

[B2] Ayano M. Kani T. Kojima M. Sakakibara H. Kitaoka T. Kuroha T. (2014). Gibberellin biosynthesis and signal transduction is essential for internode elongation in deepwater rice. *Plant Cell Environ.* 37 2313–2324. 10.1111/pce.12377 24891164 PMC4282320

[B3] Bailey-Serres J. Voesenek L. A. (2008). Flooding stress: acclimations and genetic diversity. *Annu. Rev. Plant Biol.* 59 313–339. 10.1146/annurev.arplant.59.032607.092752 18444902

[B4] Biemelt S. Keetman U. Albrecht G. (1998). Re-aeration following hypoxia or anoxia leads to activation of the antioxidative defense system in roots of wheat seedlings. *Plant Physiol.* 116 651–658. 9490765 10.1104/pp.116.2.651 PMC35123

[B5] Blokhina O. Virolainen E. Fagestedt K. V. (2003). Antioxidants, oxidative damage, and oxygen deprivation stress: a review. *Ann. Bot.* 91 179–194. 10.1093/aob/mcf118 12509339 PMC4244988

[B6] Bowler C. Van Montagu M. Inze D. (1992). Superoxide dismutase and stress tolerance. *Ann. Rev. Plant Physiol. Plant Mol. Biol.* 43 83–116.

[B7] Bradbury P. J. Zhang Z. Kroon D. E. Casstevens T. M. Ramdoss Y. Buckler E. S. (2007). TASSEL: software for association mapping of complex traits in diverse samples. *Bioinformatics* 23 2633–2635. 10.1093/bioinformatics/btm308 17586829

[B8] Byrne S. L. Nagy I. Pfeifer M. Armstead I. Swain S. Studer B. (2015). A synteny-based draft genome sequence of the forage grass *Lolium perenne*. *Plant J.* 84 816–826. 10.1111/tpj.13037 26408275

[B9] Campbell M. T. Proctor C. A. Dou Y. Schmitz A. J. Phansak P. Kruger G. R. (2015). Genetic and molecular characterization of submergence response identifies *Subtol6* as a major submergence tolerance locus in maize. *PLoS ONE* 10:e0120385 10.1371/journal.pone.0120385 PMC4373911 25806518

[B10] Chatterton N. J. Harrison P. A. Bennett J. H. Asay K. H. (1989). Carbohydrate partitioning in 185 accessions of gramineae grown under warm and cool temperatures. *J. Plant Physiol.* 134 169–179.

[B11] Chen W. Yao Q. Patil G. B. Agarwal G. Deshmukh R. K. Lin L. (2016). Identification and comparative analysis of differential gene expression in soybean leaf tissue under drought and flooding stress revealed by RNA-Seq. *Front. Plant Sci.* 7:1044 10.3389/fpls.2016.01044 PMC4950259 27486466

[B12] Colebrook E. H. Thomas S. G. Phillips A. L. Hedden P. (2014). The role of gibberellin signalling in plant responses to abiotic stress. *J. Exp. Biol.* 217 67–75. 10.1242/jeb.089938 24353205

[B13] Colmer T. D. Voesenek L. (2009). Flooding tolerance: suites of plant traits in variable environments. *Funct. Plant Biol.* 36 665–681. 10.1071/FP09144 32688679

[B14] Gallagher J. A. Cairns A. J. Thomas D. Timms-Taravella E. Skøt K. Charlton A. (2015). Fructan synthesis, accumulation and polymer traits. II. Fructan pools in populations of perennial ryegrass (*Lolium perenne* L.) with variation for water-soluble carbohydrate and candidate genes were not correlated with biosynthetic activity and demonstrated constraints to polymer chain extension. *Front. Plant Sci.* 6:864 10.3389/fpls.2015.00864 PMC4606054 26528321

[B15] Gill G. P. Wilcox P. L. Whittaker D. J. Winz R. A. Bickerstaff P. Echt C. E. (2006). A framework linkage map of perennial ryegrass based on SSR markers. *Genome* 49 354–364. 10.1139/g05-120 16699555

[B16] Gilmour S. J. Sebolt A. M. Salazar M. P. Everard J. D. Thomashow M. F. (2000). Overexpression of the Arabidopsis *CBF3* transcriptional activator mimics multiple biochemical changes associated with cold acclimation. *Plant Physiol.* 124 1854–1865. 11115899 10.1104/pp.124.4.1854 PMC59880

[B17] Guo Y. Zhu C. Gan L. Ng D. Xia K. (2015). Ethylene is involved in the complete-submergence induced increase in root iron and manganese plaques in *Oryza sativa*. *Plant Growth Regul.* 76 259–268. 10.1007/s10725-014-9996-7

[B18] Hardy O. J. Vekemans X. (2002). SPAGeDi: a versatile computer program to analyse spatial genetic structure at the individual or population levels. *Mol. Ecol. Notes* 2 618–620. 10.1046/j.1471-8286.2002.00305.x

[B19] Hsu S. K. Tung C. W. (2015). Genetic mapping of anaerobic germination-associated QTLs controlling coleoptile elongation in rice. *Rice* 8:38 10.1186/s12284-015-0072-3011-1598-4 PMC4689725 26699727

[B20] Jensen L. B. Muylle H. Arens P. Andersen C. H. Holm P. A. Ghesquiere M. (2005). Development and mapping of a public reference set of SSR markers in *Lolium perenne* L. *Mol. Ecol. Notes* 5 951–957. 10.1111/j.1471-8286.2005.01043.x

[B21] Jin Q. Wang Y. Li X. Wu S. Wang Y. Luo J. (2017). Interactions between ethylene, gibberellin and abscisic acid in regulating submergence induced petiole elongation in *Nelumbo nucifera*. *Aquat. Bot.* 137 9–15. 10.1016/j.aquabot.2016.11.002

[B22] King J. Thorogood D. Edwards K. J. Armstead I. P. Roberts L. Skøt K. (2008). Development of a genomic microsatellite library in perennial ryegrass (*Lolium perenne*) and its use in trait mapping. *Ann. Bot.* 101 845–853. 10.1093/aob/mcn016 18281692 PMC2710216

[B23] Kubik C. Sawkins M. Meyer W. A. Gaut B. S. (2001). Genetic diversity in seven perennial ryegrass (*Lolium perenne* L.) cultivars based on SSR markers. *Crop Sci.* 41 1565–1572. 10.2135/cropsci2001.4151565x

[B24] Liu M. Jiang Y. (2015). Genotypic variation in growth and metabolic responses of perennial ryegrass exposed to short-term waterlogging and submergence stress. *Plant Physiol. Biochem.* 95 57–64. 10.1016/j.plaphy.2015.07.008 26188499

[B25] Liu Q. Jiang Y. (2016). Exogenous application of nitrogen and cytokinin on growth, carbohydrate, and antioxidant metabolism of creeping bentgrass after de-submergence. *HortScience* 51 1602–1606. 10.21273/HORTSCI11357-16

[B26] Luo F. Nagel K. A. Scharr H. Zeng B. Schurr U. Matsubara S. (2011). Recovery dynamics of growth, photosynthesis and carbohydrate accumulation after de-submergence: a comparison between two wetland plants showing escape and quiescence strategies. *Ann. Bot.* 107 49–63. 10.1093/aob/mcq212 21041230 PMC3002471

[B27] Mittler R. (2002). Oxidative stress, antioxidants and stress tolerance. *Trends Plant Sci.* 7 405–410. 10.1016/S1360-1385(02)02312-9 12234732

[B28] Nelissen H. Rymen B. Jikumaru Y. Demuynck K. Van Lijsebettens M. Kamiya Y. (2012). A local maximum in gibberellin levels regulates maize leaf growth by spatial control of cell division. *Curr. Biol.* 22 1183–1187. 10.1016/j.cub.2012.04.065 22683264

[B29] Perata P. Voesenek L. A. (2007). Submergence tolerance in rice requires *Sub1A*, an ethylene-response-factor-like gene. *Trends Plant Sci.* 12 43–46. 10.1016/j.tplants.2006.12.005 17208508

[B30] Plackett A. R. G. Powers S. J. Fernandez-Garcia N. Urbanova T. Takebayashi Y. Seo M. (2012). Analysis of the developmental roles of the *Arabidopsis* gibberellin 20-oxidases demonstrates that *GA20ox1*,-*2*, and -*3* are the dominant paralogs. *Plant Cell* 24 941–960. 10.1105/tpc.111.095109 22427334 PMC3336139

[B31] Pritchard J. K. Stephens M. Donnelly P. (2000). Inference of population structure using multilocus genotype data. *Genetics* 155 945–959. 10835412 10.1093/genetics/155.2.945 PMC1461096

[B32] Rivera-Contreras I. K. Zamora-Hernández T. Huerta-Heredia A. A. Capataz-Tafur J. Barrera-Figueroa B. E. Juntawong P. (2016). Transcriptomic analysis of submergence-tolerant and sensitive *Brachypodium distachyon* ecotypes reveals oxidative stress as a major tolerance factor. *Sci. Rep.* 6:27686 10.1038/srep27686 PMC4901394 27282694

[B33] Saitou N. Nei M. (1987). The neighbor-joining method: a new method for reconstructing phylogenetic trees. *Mol. Biol. Evol.* 4 406–425. 10.1093/oxfordjournals.molbev.a040454 3447015

[B34] Sasidharan R. Mustroph A. Boonman A. Akman M. Ammerlaan A. M. Breit T. (2013). Root transcript profiling of two *Rorippa* species reveals gene clusters associated with extreme submergence tolerance. *Plant Physiol.* 163 1277–1292. 10.1104/pp.113.222588 24077074 PMC3813650

[B35] Sasidharan R. Voesenek L. A. (2015). Ethylene-mediated acclimations to flooding stress. *Plant Physiol.* 169 3–12. 10.1104/pp.15.00387 25897003 PMC4577390

[B36] Setter T. L. Laureles E. V. (1996). The beneficial effect of reduced elongation growth on submergence tolerance in rice. *J. Exp. Bot.* 47 1551–1559. 10.1093/jxb/47.10.1551

[B37] Skutnik M. Rychter A. M. (2009). Differential response of antioxidant systems in leaves and roots of barley subjected to anoxia and post-anoxia. *J. Plant Physiol.* 166 926–937. 10.1016/j.jplph.2008.11.010 19162369

[B38] Studer B. Byrne S. Nielsen R. O. Panitz F. Bendixen C. Islam M. S. (2012). A transcriptome map of perennial ryegrass (*Lolium perenne* L.). *BMC Genomics* 13:140 10.1186/1471-2164-13-140 PMC3483695 22513206

[B39] Tamang B. G. Fukao T. (2015). Plant adaptation to multiple stresses during submergence and following desubmergence. *Int. J. Mol. Sci.* 16 30164–30180. 10.3390/ijms161226226 26694376 PMC4691168

[B40] Tamang B. G. Magliozzi J. O. Maroof M. A. Fukao T. (2014). Physiological and transcriptomic characterization of submergence and reoxygenation responses in soybean seedlings. *Plant Cell Environ.* 37 2350–2365. 10.1111/pce.12277 24433575

[B41] Tamura K. Stecher G. Peterson D. Filipski A. Kumar S. (2013). MEGA6: molecular evolutionary genetics analysis version 6.0. *Mol. Biol. Evol.* 30 2725–2729. 10.1093/molbev/mst197 24132122 PMC3840312

[B42] Tamura K. Yamada T. (2007). A perennial ryegrass *CBF* gene cluster is located in a region predicted by conserved synteny between Poaceae species. *Theor. Appl. Genet.* 114 273–283. 10.1007/s00122-006-0430-z 17075706

[B43] Tan S. Zhu M. Zhang Q. (2010). Physiological responses of bermudagrass (*Cynodon dactylon*) to submergence. *Acta Physiol. Plant.* 32 133–140. 10.1007/s11738-009-0388-y

[B44] Van Veen H. Mustroph A. Barding G. A. Vergeer-van Eijk M. Welschen-Evertman R. A. Pedersen O. (2013). Two *Rumex* species from contrasting hydrological niches regulate flooding tolerance through distinct mechanisms. *Plant Cell* 25 4691–4707. 10.1105/tpc.113.119016 24285788 PMC3875744

[B45] Van Veen H. Vashisht D. Akman M. Girke T. Mustroph A. Reinen E. (2016). Transcriptomes of eight *Arabidopsis thaliana* accessions reveal core conserved, genotype- and organ-specific responses to flooding stress. *Plant Physiol.* 172 668–689. 27208254 10.1104/pp.16.00472 PMC5047075

[B46] Vashisht D. Hesselink A. Pierik R. Ammerlaan J. M. Bailey-Serres J. Visser E. J. (2011). Natural variation of submergence tolerance among *Arabidopsis thaliana* accessions. *New Phytol.* 190 299–310. 10.1111/j.1469-8137.2010.03552.x 21108648

[B47] Vashisht D. van Veen H. Akman M. Sasidharan R. (2016). Variation in *Arabidopsis* flooding responses identifies numerous putative “tolerance genes”. *Plant Signal. Behav.* 11:e1249083 10.1080/15592324.2016.1249083 PMC5157898 27830990

[B48] Voesenek L. Banga M. Thier R. H. Mudde C. M. Harren F. Barendse G. (1993). Submergence-induced ethylene synthesis, entrapment, and growth in two plant species with contrasting flooding resistances. *Plant Physiol.* 103 783–791. 12231979 10.1104/pp.103.3.783 PMC159048

[B49] Voesenek L. A. Benschop J. J. Bou J. Cox M. C. Groeneveld H. W. Millenaar F. F. (2003). Interactions between plant hormones regulate submergence-induced shoot elongation in the flooding-tolerant dicot *Rumex palustris*. *Ann. Bot.* 91 205–211. 10.1093/aob/mcf116 12509341 PMC4244986

[B50] Vriezen W. H. Hulzink R. Mariani C. Voesenek L. A. (1999). 1-Aminocyclopropane-1-carboxylate oxidase activity limits ethylene biosynthesis in *Rumex palustris* during submergence. *Plant Physiol.* 121 189–196. 10482674 10.1104/pp.121.1.189 PMC59367

[B51] Weckx S. Del-Favero J. Rademakers R. Claes L. Cruts M. De Jonghe P. (2005). NovoSNP, a novel computational tool for sequence variation discovery. *Genome Res.* 15 436–442. 10.1101/gr.2754005 15741513 PMC551570

[B52] Yang S. F. Hoffman N. E. (1984). Ethylene biosynthesis and its regulation in higher plants. *Annu. Rev. Plant Physiol.* 35 155–189. 10.1146/annurev.pp.35.060184.001103

[B53] Ye X. Q. Meng J. L. Zeng B. Wu M. Zhang Y. Y. Zhang X. P. (2016). Submergence causes similar carbohydrate starvation but faster post-stress recovery than darkness in *Alternanthera philoxeroides* plants. *PLoS ONE* 11:e0165193 10.1371/journal.pone.0165193 PMC5077152 27776170

[B54] Yu X. Bai G. Liu S. Luo N. Wang Y. Richmond D. S. (2013). Association of candidate genes with drought tolerance traits in diverse perennial ryegrass accessions. *J. Exp. Bot.* 64 1537–1551. 10.1093/jxb/ert018 23386684 PMC3617828

[B55] Yu X. Bai G. Luo N. Chen Z. Liu S. Liu J. (2011). Association of simple sequence repeat (SSR) markers with submergence tolerance in diverse populations of perennial ryegrass. *Plant Sci.* 180 391–398. 10.1016/j.plantsci.2010.10.013 21421385

[B56] Yu X. Luo N. Yan J. Tang J. Liu S. Jiang Y. (2012). Differential growth response and carbohydrate metabolism of global collection of perennial ryegrass accessions to submergence and recovery following de-submergence. *J. Plant Physiol.* 169 1040–1049. 10.1016/j.jplph.2012.03.001 22455668

[B57] Yu X. Pijut P. M. Byrne S. Asp T. Bai G. Jiang Y. (2015). Candidate gene association mapping for winter survival and spring regrowth in perennial ryegrass. *Plant Sci.* 235 37–45. 10.1016/j.plantsci.2015.03.003 25900564

[B58] Yuan L. B. Dai Y. S. Xie L. J. Yu L. J. Zhou Y. Lai Y. X. (2017). Jasmonate regulates plant responses to postsubmergence reoxygenation through transcriptional activation of antioxidant synthesis. *Plant Physiol.* 173 1864–1880. 10.1104/pp.16.01803 28082717 PMC5338657

[B59] Zhang Z. Zhang D. Zheng Y. (2009). Transcriptional and post-transcriptional regulation of gene expression in submerged root cells of maize. *Plant Signal. Behav.* 4 132–135. 19649190 10.4161/psb.4.2.7629 PMC2637500

[B60] Zhou M. Xu M. Wu L. Shen C. Ma H. Lin J. (2014). *CbCBF* from *Capsella bursa-pastoris* enhances cold tolerance and restrains growth in *Nicotiana tabacum* by antagonizing with gibberellin and affecting cell cycle signaling. *Plant Mol. Biol.* 85 259–275. 10.1007/s11103-014-0181-1 24532380

[B61] Zhou Z. de Almeida Engler J. Rouan D. Michiels F. Van Montagu M. Van Der Straeten D. (2002). Tissue localization of a submergence-induced 1-Aminocyclopropane-1-carboxylic acid synthase in rice. *Plant Physiol.* 129 72–84. 10.1104/pp.001206 12011339 PMC155872

