# Supplementary material for: Association of Candidate Genes With Submergence Response in Perennial Ryegrass
Source: Front Plant Sci. 2017 May 16;8:791. doi: 10.3389/fpls.2017.00791 (PMC5432546; doi:10.3389/fpls.2017.00791)
Supplement: Supplementary file 1 [file Data_Sheet_1.docx]

**TABLE S1** | Origin information of perennial ryegrass accessions used in this study

| ID | Accession | Origin | Status |  | ID | Accession | Origin | Status |
| --- | --- | --- | --- | --- | --- | --- | --- | --- |
| 1 | 187222 | Belgium | Uncertain |  | 83 | 284826 | Australia | Uncertain |
| 2 | 197270 | Finland | Cultivated |  | 84 | 285101 | Australia | Cultivar |
| 4 | 202451 | Argentina | Wild |  | 85 | 287849 | Spain | Uncertain |
| 5 | 204879 | Turkey | Wild |  | 89 | 303012 | UK | Cultivar |
| 7 | 206376 | Cyprus | Uncertain |  | 92 | 303026 | France | Cultivar |
| 9 | 229702 | Iran | Wild |  | 93 | 303027 | Denmark | Cultivar |
| 10 | 231569 | Libya | Uncertain |  | 95 | 636643 | Japan | Cultivated |
| 11 | 231578 | Algeria | Uncertain |  | 96 | 303037 | Sweden | Cultivar |
| 12 | 231588 | Algeria | Uncertain |  | 99 | 306292 | Bolivia | Uncertain |
| 13 | 231595 | Morocco | Uncertain |  | 101 | 317452 | Afghanistan | Wild |
| 14 | 231597 | Greece | Uncertain |  | 103 | 321397 | Czech Republic | Uncertain |
| 15 | 231605 | Portugal | Uncertain |  | 105 | 340104 | Turkey | Uncertain |
| 16 | 251141 | Yugoslavia | Wild |  | 107 | 371952 | Bulgaria | Uncertain |
| 17 | 265344 | Ireland | Cultivar |  | 108 | 376878 | New Zealand | Cultivar |
| 18 | 265351 | Chile | Uncertain |  | 109 | 384478 | Poland | Cultivar |
| 19 | 267059 | Poland | Uncertain |  | 111 | 403847 | Canada | Cultivar |
| 20 | 275660 | Australia | Cultivated |  | 112 | 403851 | Canada | Cultivar |
| 21 | 287855 | Spain | Uncertain |  | 113 | Silver Dollar | USA | Cultivar |
| 22 | 298091 | Hungary | Wild |  | 116 | 403886 | Canada | Cultivar |
| 23 | 303011 | UK | Cultivar |  | 121 | 418708 | Romania | Wild |
| 24 | 303022 | Netherlands | Cultivated |  | 122 | 418712 | Italy | Wild |
| 25 | 303031 | Netherlands | Cultivated |  | 124 | 418722 | Luxembourg | Wild |
| 27 | 403889 | Canada | Cultivar |  | 127 | 418741 | France | Wild |
| 29 | 418707 | Romania | Wild |  | 128 | 420124 | Japan | Cultivar |
| 30 | 418714 | Italy | Wild |  | 132 | Bright Star SLT | USA | Cultivar |
| 31 | 418726 | France | Wild |  | 133 | 440474 | Former Soviet Union | Wild |
| 32 | 418727 | France | Wild |  | 134 | 462335 | New Zealand | Cultivated |
| 33 | 423136 | Spain | Wild |  | 136 | 462337 | New Zealand | Cultivated |
| 34 | 462339 | New Zealand | Cultivar |  | 139 | 505840 | Former Soviet Union | Cultivated |
| 35 | 182857 | Czech Republic | Uncertain |  | 142 | 505843 | Former Soviet Union | Cultivated |
| 36 | 189392 | New Zealand | Uncertain |  | 149 | 577265 | UK | Wild |
| 37 | 198070 | Sweden | Cultivated |  | 151 | 577269 | Norway | Wild |
| 38 | 204085 | Cyprus | Uncertain |  | 153 | Manhattan4 | USA | Cultivar |
| 43 | 225825 | Denmark | Uncertain |  | 159 | 598434 | Italy | Wild |
| 46 | 231566 | Libya | Uncertain |  | 161 | 598441 | Switzerland | Wild |
| 49 | 231576 | Algeria | Uncertain |  | 164 | 598452 | UK | Wild |
| 50 | 231580 | Algeria | Uncertain |  | 166 | 598515 | Turkey | Wild |
| 55 | 231604 | Portugal | Uncertain |  | 168 | 598518 | Turkey | Wild |
| 57 | 231619 | Iran | Uncertain |  | 173 | 598911 | Tunisia | Wild |
| 59 | 234779 | Germany | Uncertain |  | 175 | Divine | USA | Cultivar |
| 65 | 251224 | Yugoslavia | Wild |  | 176 | Catalina | USA | Cultivar |
| 68 | 265336 | Sweden | Cultivar |  | 179 | 610802 | Norway | Wild |
| 72 | 265349 | Ireland | Uncertain |  | 180 | 610802 | UK | Wild |
| 77 | 274637 | Poland | Uncertain |  | 182 | 610925 | Tunisia | Wild |
| 78 | Inspire | USA | Cultivar |  | 185 | 610950 | Tunisia | Wild |
| 79 | 277846 | Yugoslavia | Uncertain |  | 187 | 611036 | Russian Federation | Wild |
| 82 | 284823 | Australia | Uncertain |  | 190 | 619474 | Romania | Cultivated |

ID number representing accessions used in this study.

Improvement status obtained from USDA germplasm bank.

**TABLE S2** | Primers used for amplification of 26 candidate genes in perennial ryegrass.

| Gene | Full name | Forward primer (5’→3’) | | Reverse primer (5’→3’) |
| --- | --- | --- | --- | --- |
| *LpACO1* | 1-aminocyclopropane-1-carboxylicacid oxidase | | GCGAGCAGAGGTTCCTGGAGTT | TGGCGATGAGCTTGGAGGTGTC |
| *LpACS* | 1-aminocyclopropane-1-carboxylic acid synthase | | GGGCGTCCTCATCACCAACC | CGAACACCACCTTCTTCCACAGC |
| *Lp6G-FFT* | Fructan: fructan 6G-fructosyltransferase | | ACCACCTCTTCTACCAGCACAAC | TCGCCGACGAAAGCCCAAAC |
| *LpGA2ox4* | Gibberellin 2-oxidase 4 | | TGCGAGCGGTTCGGGTTCTT | TGCCGTTGGAGTGCAGGATG |
| *LpGA20ox* | Gibberellin 20-oxidase | | GAGATCATGGAGGTGCTGGGTG | TTATGTCGTCGTCGTCGTGGC |
| *LpPDC* | Pyruvate dehydrogenase E1 component subunit alpha-3 | | GGCTGTATCCACTGGCTTCAT | GAGTTGGTCCTTCACCTCTCC |
| *Lp6-SFT* | Sucrose: fructan 6-fructosyltransferase | | GCTTCTGATGTTGCTGCCA | GAGTCCTTAACCATGACGGTCT |
| *LpLDH* | L-lactate dehydrogenase | | ATGCACAAGACGTCATCGCTGTC | CTGGGCATTGACGTCGAGGTG |
| *Lp1-SST* | Sucrose: sucrose 1-fructosyltransferase | | CTCCGCGAATGGATCAAGC | CCGATGTATGCCCAGACGAT |
| *LpCAT* | Catalase | | TGTTCDCCTTCCTCTTYGACGAT | GTGGTGGTTGTTGTGGTATGC |
| *LpCBF3b* | C-repeat binding factor 3b | | TACCTGCACCAAGCGATCTA | AGGTTTCTCGTCGATCGTTACT |
| *LpCBF1b* | C-repeat binding factor 1b | | CTCACAGTCCACAGTCCACC | GAACTGCATCTGCTTGCATG |
| *LpCBF3c* | C-repeat binding factor 3c | | TACCTGCACCAAGCGATCTA | CGACAGTGACGTCAGTAGGTCCGT |
| *LpCBF4b* | C-repeat binding factor 4b | | TTCACCGGGCATCTCCACTAG | CCGTTGCATCATAAGTTGCA |
| *LpChlCu-Zn SOD* | Chloroplastic copper-zinc superoxide dismutase | | TCGYGGACGCYACTAAGAAG | CAACAACACCACATGCCAGTCT |
| *LpCyt Cu-Zn SOD* | Cytosolic copper-zinc superoxide dismutase | | TTRCTAGCAGTGAGGGTGTCAA | ATGATTCCGCAAGCAACTCGG |
| *LpDHAR* | Dehydroascorbate reductase | | CTCGGCGACTGYCCATTCWC | TTTGGTGCCCATCCAGCAAT |
| *LpFeSOD* | Iron superoxide dismutase | | GCBCTCACTACTCCKCCGTAT | TGCGTAGAGTGACAGTATCCCA |
| *LpGPX* | Glutathione peroxidase | | GGTAACGATGTGAGTTTGAGCC | GTCCCARCAGAGCTTGAATRTC |
| *LpGR* | Glutathione reductase | | CCAAGGTYGCGATCTGCGAG | GCAACAGCAATGCCCTGCATAA |
| *LpLEA3* | Late embryogenesis-abundant, group 3 | | ATGGCTTCTTCTTCCGTGCTGCT | TACTCTTTGGCCGCGGCTTT |
| *LpMAPK* | Mitogen activated protein kinase | | TATATCCGCCAACTTCCCCG | TCAATCATCACCTGCCCACC |
| *LpMDHAR* | Monodehydroascorbate reductase | | AGCTCTGCATCATCTCCGAA | ACTGCCAGGACAATGTGAAGAC |
| *LpMnSOD* | Manganese superoxide dismutase | | ATGGCBCTCCGCACGYTG | CCGTTGAACTTGATGGCGCTCT |
| *LpPIP1* | Plasma membrane intrinsic protein, type 1 | | ACCTTCCTCTTCCTCTACGTGACCAT | AGGTGTGCTCCCTGTTGTAGATGA |
| *LpTIP1* | Tonoplast intrinsic protein, type 1 | | TCATCTCCACGCTCATCTTCGTCT | CGACCCAGTACACCCACTGGTAT |

**TABLE S3** | Association of candidate genes with traits under control (C) in 94 perennial ryegrass accessions.

| Putative gene | Traits | SNP (bp) | Allele | *P*-value | Model |
| --- | --- | --- | --- | --- | --- |
| *Lp1-SST* | C-Color | 972 | C:CG:G | 3.25E-04 | Q+K |
|  | C-LFW | 871 | T:/GT | 7.61E-05 | Q+K |
|  | C-LDW | 871 | T:GT | 8.24E-05 | Q+K |
|  | C-LDW | 1,146 | A:AG:G | 2.87E-04 | Q+K |
| *Lp6G-FFT* | C-Color | 835 | G:GA:A | 1.86E-04 | Q+K |
| *LpCBF1b* | C-HT | 267 | C:CT:T | 2.91E-04 | Q |
|  | C-LDW | 441 | C:T | 1.01E-09 | Q+K |
|  | C-LDW | 282 | C:CG:G | 3.51E-05 | Q+K |
| *LpACO1* | C-LFW | 460 | C:CG:G | 9.65E-05 | Q+K |
|  | C-LFW | 469 | C:CT | 1.05E-04 | Q+K |
|  | C-LDW | 460 | C:CG:G | 1.14E-04 | Q+K |
|  | C-LDW | 469 | C:CT | 7.45E-05 | Q+K |
| *LpFeSOD* | C-Fv/Fm | 333 | A:CA:C | 2.82E-04 | S |
|  | C-Fv/Fm | 207 | C:CG:G | 7.83E-04 | S |
| *LpACS* | C-Color | 981 | A/:AG | 8.81E-04 | Q+K |
| *LpCyt Cu-Zn SOD* | C-HT | 269 | A:AT:T | 9.69E-04 | Q |

**
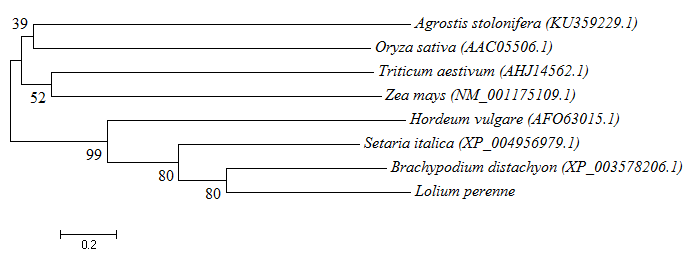
**

***ACO1***

***6G-FFT***

**
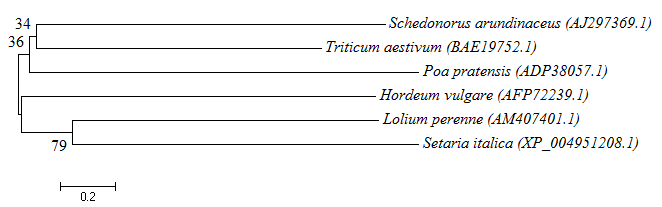
**

**Fig. S1**. Phylogenetic tree of 1-aminocyclopropane-1-carboxylicacid oxidase (ACO1) and fructan: fructan 6G-fructosyltransferase (6G-FFT) from plant species. NCBI protein accession numbers were use d to indicate homologous protein.
